# Towards long double-stranded chains and robust DNA-based data storage using the random code system

**DOI:** 10.3389/fgene.2023.1179867

**Published:** 2023-06-13

**Authors:** Xu Yang, Xiaolong Shi, Langwen Lai, Congzhou Chen, Huaisheng Xu, Ming Deng

**Affiliations:** ^1^ Institute of Computing Science and Technology, Guangzhou University, Guangzhou, China; ^2^ College of Information Science and Technology, Beijing University of Chemical Technology, Beijing, China

**Keywords:** DNA-based data storage, long double-stranded chains, random matrix, random equilibrium, highly robust, random code system

## Abstract

DNA has become a popular choice for next-generation storage media due to its high storage density and stability. As the storage medium of life’s information, DNA has significant storage capacity and low-cost, low-power replication and transcription capabilities. However, utilizing long double-stranded DNA for storage can introduce unstable factors that make it difficult to meet the constraints of biological systems. To address this challenge, we have designed a highly robust coding scheme called the “random code system,” inspired by the idea of fountain codes. The random code system includes the establishment of a random matrix, Gaussian preprocessing, and random equilibrium. Compared to Luby transform codes (LT codes), random code (RC) has better robustness and recovery ability of lost information. In biological experiments, we successfully stored 29,390 bits of data in 25,700 bp chains, achieving a storage density of 1.78 bits per nucleotide. These results demonstrate the potential for using long double-stranded DNA and the random code system for robust DNA-based data storage.

## 1 Introduction

In the age of information, the volume of data is exponentially increasing, driving the need for more efficient storage devices. DNA, as the storage medium of organisms, has been naturally selected through billions of years of evolution. Its double-stranded structure is highly stable and can efficiently perform the functions of replication, retrieval, and transcription of information under the catalyst of biological enzymes. Scientific research has shown that DNA has a storage density of approximately 10^19 bits per cubic centimeter, which is significantly higher than that of electronic storage devices (Fontana and Decad, 2014; Extance, 2016) (Semiconductor Industry Association, 2020). Moreover, DNA requires very little energy for long-term storage (Orlando et al., 2013). (Dong et al., 2020). However, the high cost of DNA synthesis and sequencing remains a major challenge for molecular storage (Antkowiak et al., 2020). Nevertheless, as technology advances, the cost of DNA storage is rapidly decreasing, making it an increasingly viable option for next-generation data storage.

The feasibility of large-scale DNA storage was first demonstrated by Church in 2012 (Church et al. 2012). The proposed coding scheme mapped two bases to one binary bit for data storage, where A or C was mapped to binary 0, and G or T were mapped to 1. Although the storage information density reached only 0.83 bits per nucleotide, the proposal marked the beginning of DNA storage research. Later, Goldman et al. (Goldman et al., 2013) utilized quadruple redundancy to achieve reliable DNA storage, but this reduced the information storage density due to excessive redundancy. In recent years, scholars have developed finely designed coding schemes to obtain the maximum information capacity while satisfying biological constraints. Furthermore, different molecular strategies have been proposed to increase the logical density of DNA storage, such as using the distribution of base content or chemically modified DNA nucleotides (Tabatabaei et al., 2022) (Anavy et al., 2019). However, chemically modified DNA nucleotides require precise synthesis and sequencing, increasing the cost and introducing more instability cases.

Currently, the main method of DNA storage is still using basic transcoding rules (i.e., converting [A, T, C, G] to [00, 01, 10, 11]). With these rules, Erlich et al. (Erlich and Zielinski, 2017) reported a coding strategy called DNA Fountain in 2017, which demonstrated a theoretical coding potential of 1.98 bits per nucleotide. The fountain code technique is widely used in DNA storage due to its ratelessness and the ability to pre-screen biological constraints. Biological constraints limiting DNA storage typically refer to homopolymers (consecutive repeating bases), GC content (the ratio of the number of G or C bases in the DNA strand to the total number of all bases), and micro-satellites (short tandem repeats). According to Schwartz et al. (Schwartz et al., 2012), when the homopolymer exceeds 4, the probability of insertion and deletion errors in the DNA strand increases significantly during Illumina sequencing analysis. Ananda et al. (Ananda et al., 2013) showed that when the homopolymer exceeds 4, the error rate during PCR amplification, synthesis, and sequencing also increases. Schwartz et al. (Schwartz et al., 2012) reported that high (above 0.55) or low (below 0.45) GC content would lead to an increase in error rate during PCR. When micro-satellites are present in the DNA strand, interfering term errors are generated during PCR, which can cause unstable DNA structures.

In DNA data storage, many different approaches can be used to encode data into codewords. DNA coding is a key step in DNA storage and can directly affect storage performance and data integrity. However, since errors are prone to occur in DNA synthesis and sequencing, and non-specific hybridization is prone to occur in the solution, how to effectively encode DNA has become an urgent problem to be solved (Xiaoru and Ling, 2021).

The LT encoder’s degree selected by the robust soliton distribution is small, it is not guaranteed that all K sub-packets will be encoded through (1+ε)*K coding (where ε is the redundancy coefficient and K is the number of segment packets obtained by dividing the original data) (Schwarz and Freisleben, 2021). Moreover, the vast majority of data generated by the fountain code encoder cannot be screened by biological constraints, which reduces the encoding efficiency. The base 64 encoder (Zhang et al., 2020) can satisfy the GC content and homopolymer, but reduces the encoding and storage density because of it is balance code. GCNSA can construct a larger set of non-data bits under the same DNA sequence length and coding conditions, and can address more DNA sequences with fewer bases, thus improving the density of DNA storage. To sum up, GCNSA is mainly used for non-data bit encoding (Cao et al., 2023). The MFOL decoder is programmed to construct the DNA storage codes by reducing the error rates of DNA coding sets with GC-content, Hamming distance, and No-runlength constraints (Rasool et al., 2021).

In order to improve the reliability and efficiency of the encoder, we propose a random code (RC) system inspired by random matrix theory and the pseudo-random number generators of electronic computers. The RC system consists of three main components: random matrix, Gaussian preprocessing, and random equilibrium. Firstly, we use a pseudo-random number generator to create a random matrix, which is then subject to Gaussian preprocessing using the XOR elimination algorithm. This preprocessing step results in a generated matrix, which is a submatrix of the original random matrix with optimal decoding success rates. This ensures that all chunks of data are included in the generated matrix. Additionally, we propose a random equilibrium algorithm to ensure that the generated DNA sequence can pass the biological constraints screening successfully. The random equilibrium algorithm is applicable to any file format, even those with extremely high 0/1 rates (such as more than 80% consecutive 0s or 1s).

To demonstrate the compatibility of the RC algorithm, we successfully stored a 29,390-bit.txt document in a 25700bp plasmid double strand using our system. Our experimental results confirm that the RC system has excellent robustness and reliability, as well as high information storage density. In fact, our biological experiments verified that the storage density of RC is above 1.78 b t/nt.

## 2 Materials and methods

### 2.1 RC algorithm steps

(1) Splitting the target storage document into K sub-packets based on the data capacity of the generated DNA strands.
(2) Using adapter as seeds injected into a pseudo-random generator to generate 0/1 random matrices of specified dimensions.
(3) Gaussian preprocessing is performed on random matrices with Gaussian XOR elimination, and select the generated matrix.
(4) Based on the generated matrix, which labels the chunks involved in the XOR operation according to the elements are 1, and thus generates the droplet.
(5) Random equilibration of data based on biological constraints such as homopolymer and GC content, to obtain the final storable DNA strand.

The DNA strand in our experimental validation consisted of the following main components. For more encoder steps, see the Encoder section in the Supplementary Material.

Adapter (20 nt): as pseudo-random number generator seeds, which can also be used for information retrieval and PCR amplification primers.

Times (6 nt): used to record the number of times the pseudo-random number generator generates a random matrix.

Data payload (639 nt): used to store the droplet after the generated matrix guide, after the corresponding chunks are XOR.

XOR Equilibrium (10 nt): Make the DNA strand meet the biological constraints.

XOR re-equilibrium (2 nt):same as XOR Equilivrium.

**FIGURE 1:**
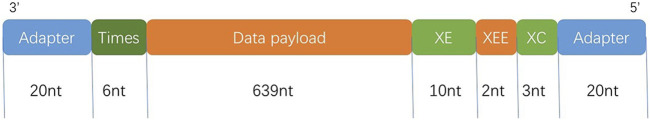
Structure of the oligos. Black labels, length in nucleotides. 5′ is the phosphoric acid group of DNA, and 3′ is the hydroxyl group.

XOR Check (3 nt): Bitwise XOR of the whole chain (as Figure 1 shows).

### 2.2 Random matrix degree distribution function

In the random matrix, each row has K elements, the elements can choose to take the value of 0 or 1 (each with 50% probability), and a single element to take the value of the binomial distribution (*p* = 0.5). If the probability distribution of the random matrix degree, that is, the probability distribution of the sum of the vector elements of each row of the matrix for 1, the distribution function conforms to the normal approximation of the binomial distribution, noted as X ∼ N (K/2, K/4), and its probability density function is:

$$F\left(x\right)=\frac{n\pi x}{\sqrt{2\pi }\sigma }e^{\left(-\frac{{\left(x-\mu \right)}^{2}}{2\sigma^{2}}\right)},$$ (1)

where variance 𝜎2 = K/4 and the mathematic expectation μ = K/2.

Gaussian preprocessing s are assumed to partition the original data into K copies. The matrix size can be solved by reaching K*K dimensions. However, to cope with the possible errors in DNA synthesis and storage sequencing, logical redundancy needs to be added. Assuming m strands are added as redundancy, the final coded DNA strands reach (K + m). The degree distribution matrix reaches (K + m)*K dimensions. Among the (K + m) strands, K strands are randomly selected to form the K*K dimensional degree distribution matrix with maximum probability solvable, which becomes the focus of our study. Since the degree distribution matrix operation is a XOR or operation, not a linear system of equations solution in the traditional sense, it is not possible to use the coefficient matrix as a non-singular matrix as the only determination condition for the matrix solvability. We here use Gaussian heteroskedastic elimination variation (the algorithm complexity is K3) to construct it as a triangular matrix. If all n elements of the main diagonal are 1, then the system of equations has a unique solution. If 0 exists in the first n elements of the main diagonal, then the system of equations has no unique solution.

We utilized singular value decomposition, random sample, and correlation alignment for comparison. It is finally found that the solution solved using random sampling is optimal within a certain constraint time to achieve the maximum success rate of sample decoding. Stochastic equilibrium in the RC algorithm.

When the stored DNA strand length increases, the randomly generated water droplet data will be more difficult to pass the screening, taking the homopolymer condition as an example, assuming that the probability of occurrence of homopolymers is expressed as $Q\left(m,l\right)$ .

$Q\left(m,l\right)$ is the probability to observe up to an m-nt homopolymer run in a random l-nt sequence.

It is assumed that m homopolymers occur at completely random locations and the corresponding probability distribution conforms to the binomial distribution. P denotes the probability of occurrence of m homopolymers and q = 1-p, the probability of non-occurrence of homopolymers. According to Feller (WilliamFellerWrited, 1958) et al. the probability can be approximated as

$$q_{m}\left(p,l\right)\approx \frac{\beta }{x^{l+1}},$$ (2)

where x is:

$$x=1+q\times p^{m}+\left(m+1\right)\times {\left(q\times p^{m}\right)}^{2},$$ (3)

and β is:

$$\beta =\frac{1-p*x}{\left(m+1-m*x\right)*q}.$$ (4)

According to previous studies, for practical purposes, the probability distribution of Q, which can be approximated by $q_{m+1}$ , and we approximate the distribution of observing up to m-nt homopolymer runs as the product of four independent events:

$${Q\left(m,l\right)\approx \left[q_{m+1}\left(p=0.25,l\right)\right]}^{4}.$$ (5)

According to the formula it is seen that when m = 4 and l = 700, Q = 0.025%, which means that when the length of the synthesized DNA strand is 700 nt, the probability of randomly synthesizing each strand with the presence of more than 4 base repeats is: 99.75%.

To optimize these problems, Abdur Rasool et al. (Rasool et al., 2023) proposed a computational evolutionary approach based on a synergistic moth flame optimizer (MFO), which took the Levy flight and opposition-based learning mutation strategies by constructing reverse-complement constraints. However, with the expansion of the data scale, it will become extremely difficult to solve the MFO matrix.

### 2.3 Random equilibrium

We address this situation using a random equilibrium approach. Our idea is to focus on chains that do not pass the biological constraint filter, and the vast majority of randomly generated chains during encoding fail to pass the biological condition filter. This keeps cycling the generation of random chains, which reduces the coding efficiency. If the chains are actively trained to satisfy the constraints, the coding efficiency will be greatly improved. We first inject the seed (adapter) into the random number generator. By randomly generating random bases with the same length as the target chain, the generated random bases and the target bases do the heteroskedastic operation to play the role of equalization, and through multiple equalization to achieve the purpose of meeting the biological constraints. After simulation experiments, it was found that the training of 25 pieces of 645 nt data information can be completed by 10 nt of random equalization space, so that 100% of them pass the biological condition screening (homopolymer<4, GC content 45%–55%). In the biological experimental validation, we set up 10 nt of XOR equalization sites. The GC content, homopolymer and minimal satellites (Micro-satellites) of the DNA strand were first used as screening conditions. If the DNA strand fails the screening, a random DNA strand is generated by a pseudo-random number generator. This random DNA strand has the same length as the target DNA strand. The two strands are subjected to a XOR operation. After the operation, the result of the dissimilarity is again judged by the constraint. If it is not satisfied, the pseudo-random number generator is used again to generate a random base sequence for the hetero-or equalization until the constraint is satisfied or the coding space of the pseudo-random number generator is exhausted. If a strand satisfying the constraint is generated, the number of times the pseudo-random number generator is generated is recorded in the XOR equalization bit and biosynthesized with the overall DNA data information.

### 2.4 Decoding steps

When decoding, first sequencing the nucleotides, finding the forward and reverse primers according to the sequencing results, screening to get the target DNA chain sequence, and judging whether insertion and deletion errors occur according to the length of the chain; after that, according to the function of each part of the DNA chain, reducing the information of the DNA chain; according to the Times information, using Adapter as the seed, using the pseudo-random number generator to generate a random matrix Then, add Data payload as the augmentation matrix and solve the matrix using Gaussian XOR elimination method to get the original data information. For more details of the decoder, please check Encoder in the Supplementary Material.

The Gaussian XOR elimination method (Schreiber, 1982) used in this decoding method will traverse each row of data in the matrix, and the rows with related information will be XORed, which can effectively avoid the problem of missing information XOR in fountain code BP decoding. At the same time, the XOR Check bit of 3 nt is set, and the target data can be detected in units of 3 nt. When it is found that the information does not match, the error chain can be effectively eliminated to prevent the error chain from disturbing the calculation during the decoding process.

### 2.5 Experimental material

The experimental material used is PUC57 plasmid, which has the advantages: Firstly the length can be in the range of 50-1.5 k Bp. Longer strands mean that more data can be stored without wasting too many common primers and search addresses, which will make the storage density higher; Secondly in the form of double-stranded loops, the structure is more stable, avoiding the formation of some secondary structures and having better robustness. Thirdly it can be implanted into living organisms, allowing more efficient and low-cost intra-organismal replication.

## 3 Results

### 3.1 The general principle and features of the RC

A fountain can be seen as the circulation of a large number of droplets. When we need a glass of water, we simply fill it up at the fountain, without caring which droplet is actually used. Fountain codes function well for electromagnetic communication because the communication is synchronous between the transmitter and receiver, thus giving the information source a chance to send more data packets for successful data recovery (Ping et al., 2022). However, DNA-based data storage is heterochronic, so it is necessary to ensure the information we synthesize has an optimal decoding success rate.

The general principle of RC is to guarantee the droplets achieved the optimal decoding effect. It overcomes the inherent problems of LT codes, improves the coding efficiency. We can use the pseudo-random number generator to get the random matrix fleetly. Then got the generated matrix by Gaussian preprocessing, and this matrix can generate the droplets which have the optimal decoding success rate. Random equilibrium prevents information from being discarded, so that the data can smoothly pass the screening of biological constraints and thus be stored for a long time.

**FIGURE 2:**
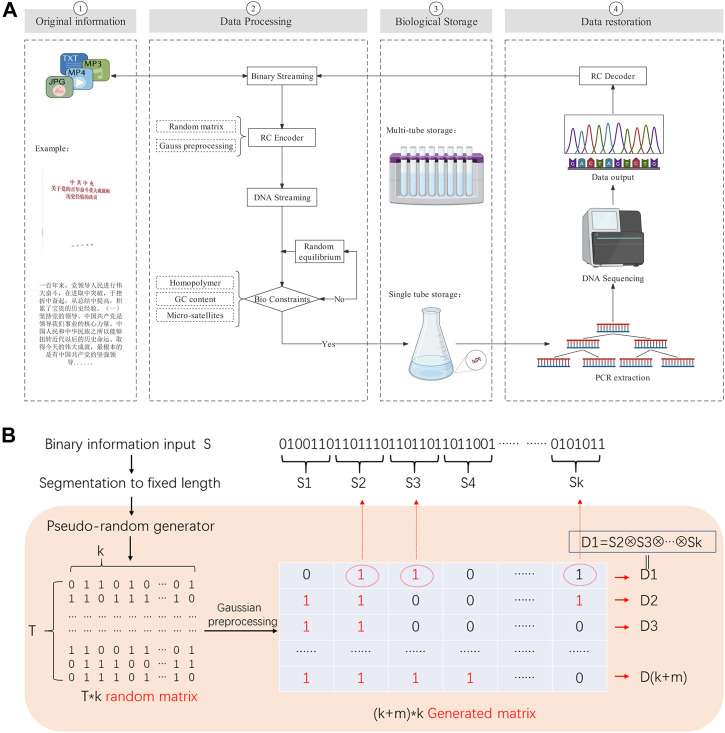
**(A)** DNA storage flow chart. Our experiment stored the historical experience of the Communist Party of China’s century-old struggle, the full text of 2,927 Chinese characters. The binary file is converted into DNA data by RC encoder, and then synthesized the DNA which meet the biological constraints by random equilibrium. It can be recovered into binary file data by PCR and sequencing. **(B)** the RC decoder flow chart. We use the pseudo-random number generator to get the generated matrix, it labels the chunks involved in the XOR operation according to the elements are 1, and thus generates the droplet.

The basic process of DNA storage we designed is to first perform binary extraction and segmentation of the files to be stored. Next, the binary information is transcribed into base sequences that satisfy the biological constraints by the RC encoder. Then the base sequences are biologically synthesized and stored. When the information is required, the DNA is sequenced and the information is recovered by a decoder (as Figure 2A shows).

The basic algorithm of RC encoder is: Firstly, the specified seeds are injected into the pseudo-random number generator to generate a 0/1 random matrix of T*k dimension (T depend on the random space capacity, k is the number of segment packets); Secondly, Gaussian preprocessed the random matrix to get the generation matrix of (k + m)*k dimension (m is the redundancy) with the optimal decoding rate. Finally according to the generation matrix, got the droplets (as Figure 2B shows).

### 3.2 The degree of RC

In the coding scheme of fountain codes, the degree, which refers to the number of sub-packets involved in coding, is a critical parameter. If the degree is too high, it leads to increased correlation among information sub-packets, resulting in higher coding complexity. On the other hand, if the degree value is too low, there is a higher probability of sub-packet loss during transmission due to the low participation rate of sub-packets in coding.

**FIGURE 3:**
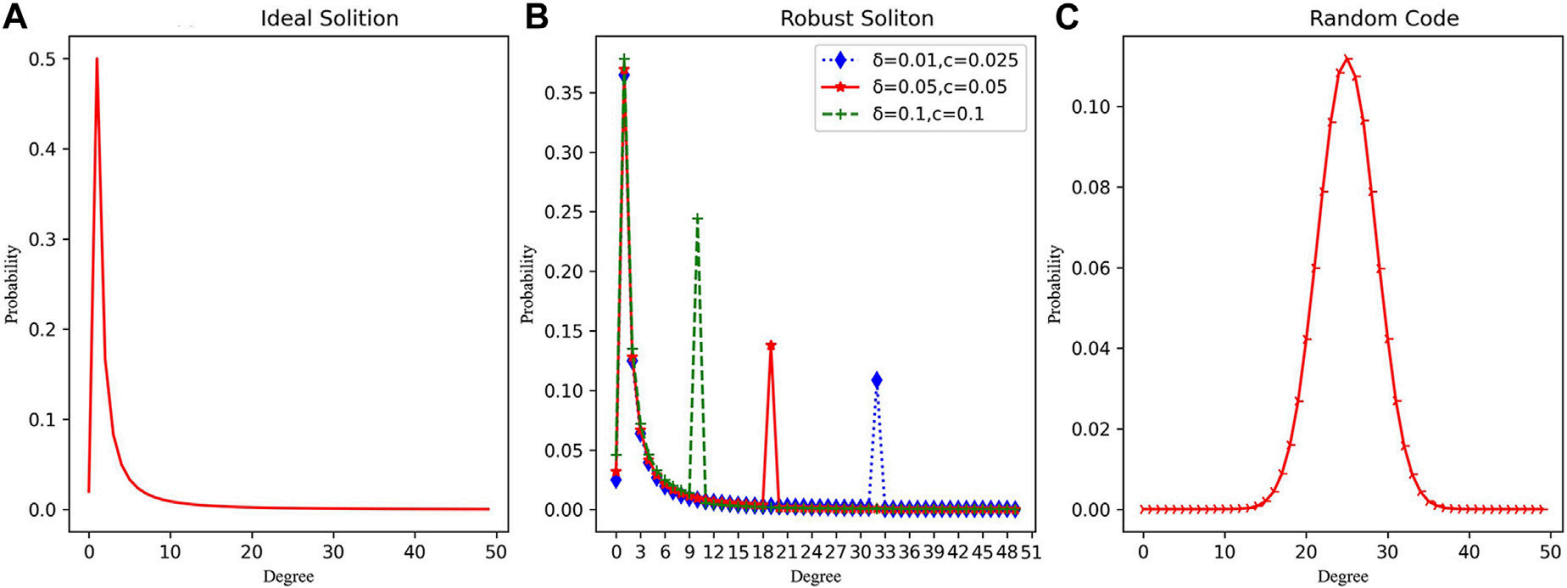
The degree distribution curves of different coding methods. **(A)** Ideal soliton’s degree distribution. **(B)** Robust soliton’s degree distribution with different parameters. **(C)** Random code’s degree distribution.

When compared to LT codes, it is evident that the degree of the RC system is sampled in the form of a normal distribution, with a variance of σ2 = K/4 and an expectation of μ = K/2 (as Figure 3 shows). The mean value of the degree is higher, indicating that the RC system possesses stronger information relevance and redundancy compared to LT codes.

### 3.3 Random equilibrium

To ensure that the generated data meets biological constraints, it needs to be screened before biochemical synthesis of DNA molecules. One way to achieve this is by using a pseudo-random number generator to obtain multiple random sequences that are as long as the droplets. Then, XOR operations can be performed between the random sequences and the droplets to increase the probability of the droplets satisfying the biological constraints. After simulation experiments, it was found that the training of 25 pieces of 645 nt data information can be completed by 10 nt of random equalization space, so that 100% of them pass the biological condition screening. In the biological experimental validation, we set up 10 nt of XOR equalization sites. This approach can help to reduce the error rate during synthesis, storage, PCR, and sequencing.

### 3.4 Data recover

Errors such as deletion, insertion, and substitution can occur during DNA storage, synthesis, PCR, or sequencing. Among these errors, insertion and deletion errors at a single site can alter the overall length of the DNA strand and significantly affect the robustness of DNA storage and decoding success rate. To address these errors, researchers commonly add error-correcting codes such as Reed-Solomon (RS) code, low-density parity-check code (LDPC), or checksum Recovery Correction (CRC). However, these methods have limited roles and can only ensure error recovery within a certain range. Moreover, they cannot correct insertion and deletion errors (Lenz et al., 2019). In fountain code, if the error is not corrected or identified, the file cannot be recovered (Zhang et al., 2021). Zihui Yan et al. (Yan et al., 2022a)^.^ (Yan et al., 2022b) proposed a DNA error correction method called DNA segment Levenshtein-Marker (DNA-LM). The codeword length computing complexity is constrained in linear time. But DNA-LM increases the redundancy and reduces the information coding density.

**FIGURE 4:**
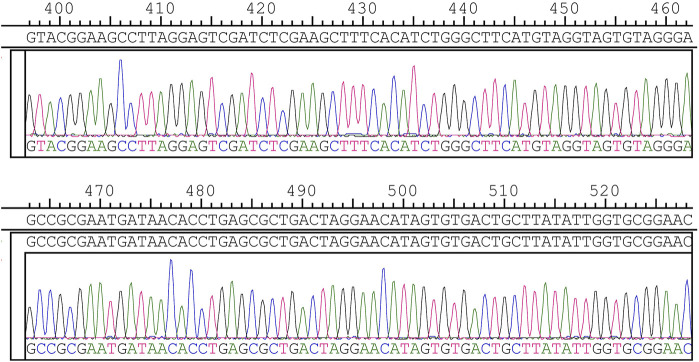
700bp storage information Sanger sequencing partial results.

Thus, logical redundancy must be set up to ensure error tolerance. In DNA sequencing, third-generation sequencing has high throughput sequencing capability and low accuracy. Qu G et al. (Qu et al., 2022) analyzed the sequencing results using the Clover clustering method, which shows quickly and accurately clustered the sequencing results. In this study, we used the solid-phase phosphoramidite triglyceride method for accurate DNA synthesis and Sanger sequencing for high sequencing accuracy (as Figure 4 shows), which is known as the golden key to DNA sequencing (邱超孙含丽,, 2008). Based on the reliable synthesis and sequencing technology, we used the XOR check method to perform error checking and achieved the best detection efficiency.

**FIGURE 5:**
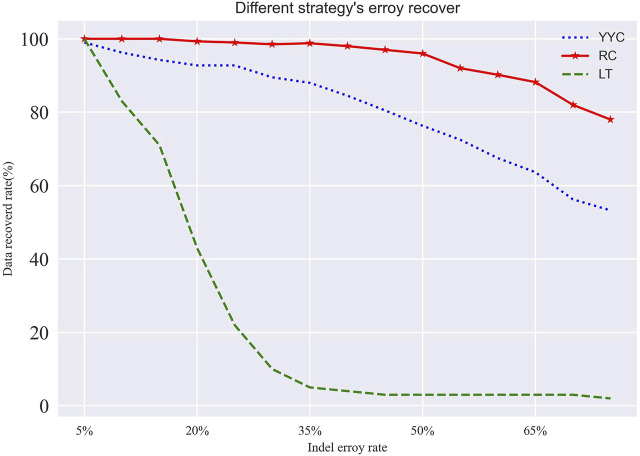
Recovery rate of different encoding methods in case of insertion and deletion errors. Where the logical redundancy additions are 25%.

Based on the error correction results, the decoding success rate of RC is higher than that of LT and YYC within the logical redundancy coverage when it comes to insertion, deletion, and loss errors (as Figure 5 shows). This is because RC has a larger degree average, allowing it to cover most of the sub-packet information with minimal logical redundancy. Consequently, RC has a higher information correlation, and its decoding success rate drops sharply when the error rate exceeds the logical redundancy range.

Here, we stored a summary of the historical experience of the 19th Central Committee of the Communist Party of China, which was adopted at the 6th Plenary Session of the 19th Central Committee of the Communist Party of China. The summary contained a total of 2,939 characters, which were transcoded into 29,390 bits. We used RC to generate 25 chains of 700 base pairs, including adapters, resulting in a storage density of 1.78 bits per nucleotide. In this experiment, we used K = 23, and added m = 2 redundant chains.

For our biochemical experiments, we designed 20 nt pre- and post-adapters for use with 660 nt data information. We chose the PUC57 plasmid as the DNA storage vector and ampicillin for bacteriophage sample resistance. The sequencing primers M13 F/R were used for sequencing. Firstly, we synthesized the designed DNA sequence, then we cleaved the plasmid at a specific site using restriction endonuclease, added the synthesized target sequence to the plasmid, and finally sequenced using the Sanger method. If any errors were found in the sequencing result, we repeated the synthesis process until the sequencing result was correct. We then performed PCR amplification and dried the samples into powder for long-term storage.

To validate the coding scheme, we dissolved the synthesized sample powder in pure water to obtain a sample content of 50 mol/μL. We then sent the samples to two different companies, Wuhan AuGCT DNA-SYN Biotechnology Co., Ltd. and Shenggong Bioengineering (Shanghai) Co., Ltd., for sequencing verification. Wuhan AuGCT DNA-SYN Biotechnology Co., Ltd.’s sequencing results showed no errors and could be decoded correctly. However, Shenggong Bioengineering (Shanghai) Co., Ltd.'s sequencing results had one base substitution error in one strand as confirmed by XC verification. Despite this error, the data information could still be recovered normally after deleting the affected strand. A comparison of the experimental results is presented in the following Table1. Table 2 shows the simulation results of different storage file formats.

**TABLE 1:** Comparison of DNA-based storage coding schemes.

Refs	Length (nt)	Bits per base including primers	Bits per base excluding primers	Random access	Coverage	Code	Contents	Storagecapacity
Church et al. (2012)	115	0.6	0.83	No	3,000×	1 bit to 1 base	English text, JPG images, computer code	650 KB/630 KB
Goldman et al. (2013)	117	0.19	0.33	No	51×	Rotating encoding	Text file, JPEG file, MP3 file	739 KB
Grass et al. (2015)	158	0.86	1.14	No	372×	Reed–Solomon coding	Text from the Swiss Federal Charter	83 KB
Organick et al. (2018)	150–200	0.81	1.1	Yes	4–11×	Reed–Solomon coding	high-definition video, images, audio, and text	200.2 MB/33 KB
Bornholt et al. (2017)	120	0.57	0.85	Yes	40×	rotating encoding	Three JPG files	151 KB
Erlich and Zielinski (2017)	152	1.18	1.57	No	10.5×	DNA fountain encoding	Text file, SVG file, Video file	2 MB
Jeong et al. (2021)	152	1.17	1.53	No	600×	DNA fountain encoding	JPG file	513.6 KB
Choi et al. (2020)	85	1.78	3.37	No	250×	One character	Text file	854 B
Anavy et al. (2019)	152	-	1.57	No	-	Standard Σ4 +DNA-level	zip file bilingual Bible	6.42 MB
						Reed–Solomon + Fountain		
			1.76			Composite Σ5 + DNA-level		
						Reed–Solomon + Fountain		
			1.96			Composite Σ6 + DNA-level		
						Reed–Solomon + Fountain		
Zhang et al. (2020)	834	1.77	1.96	No	-	Base 64	Text file	185B
Ping et al. (2022)	200	-	1.75–1.78	No	-	YYC +	.jpg and.txt	1 GB
						Reed–Solomon coding		
Cao et al. (2022)	162	1.29/1.22	1.41	Yes	35×	DNA constraint + fountain encoding + RS	Mp3, mp4, txt, jpg, pdf files	480 KB/83.3 KB
This work	660	1.68	1.78	No	-	Random code	Mp3,mp4, txt, jpg, pdf files	8.805 KB

**TABLE 2:** RC’s simulation results of different storage file formats.

File name	File data format	Storage space	Number of chains	Length of a chain (nt)	redundancy (%)	Bits per base including primers
the sound of silence	.mp3	3072 Kb	20,357	700	3	1.72
Mona Lisa Smile	.jpg	6757 KB	43,309	700	3	1.77
The founding of the People’s Republic of China	.mp4	44775 KB	287,003	700	3	1.77

The experimental results demonstrate that the RC system achieves higher logical density and stronger information recovery compared to the LT code. The RC system has also shown significant improvements in sub-packet selection and error recovery, meeting the requirements of adequate and reliable sub-packet capture. Furthermore, the random equalization technique used in the RC system satisfies biological constraints, making it an effective solution for DNA data storage.

## 4 Discussion

### 4.1 DNA long chain storage

As genetic material, DNA exists naturally in the form of long double-stranded chains. For example, in humans, the 24 pairs of chromosomes contain varying lengths of base pairs, with the first chromosome containing 249,250,621 bp and the shortest chromosome, the 21st, containing 48,129,895 bp. The base complementary pairing in DNA duplexes is stable and provides high replication and transcription efficiency. Additionally, the double-stranded structure provides a natural backup under the rule of complementary pairing, making information storage more reliable. Therefore, the use of longer DNA double strands as carriers is the future direction of DNA storage. Wang, P. H. et al. Compressed the address information to increase the information part (Wang et al., 2022). Lin K N et al. used T7 promoter to generate longer sequences, but the complexity of the experiment is increased due to additional transcription and reverse transcription processes (Lin et al., 2020).

As shown in Table 1, most current biological storage media use single-stranded oligonucleotide sequences with chain lengths ranging from 100 to 200 nt. For example, in 2020, Zhang Y (Zhang et al., 2020) stored 171 English letters and symbols (185 Bytes) in a DNA double strand of 834 B P using the Base 64 code table and loaded it into pGH-plasmid. However, due to the limitation of the code list, punctuation, and special characters cannot be stored, and its generality is limited. Moreover, Zhang Y (Zhang et al., 2020) only stored one strand, without any addressing, retrieval, or error correction functions. In this paper, we propose a method that uses 25 DNA double strands of 700bp to perform multiple functions, such as compiling, storing, retrieving, and error-checking information, thereby opening up a new coding method for DNA long-strand storage. As the length of DNA sequences increases, their biological constraints become more stringent. Cases of homopolymer and sequence duplication occur more frequently, and balancing local GC content becomes more difficult. To address these issues, this paper proposes a random equilibrium approach that provides a unique and efficient solution for screening long DNA sequences against biological constraints.

### 4.2 Degree of the fountain code

In the coding scheme of fountain codes, the selection of sub-packets is a critical factor that affects the success rate of decoding. LT codes (Luby, 2002) select the degree by robust isolated sub-distribution, while Maymounkov (2002) use an Online codes technique to increase the probability of sub-packet participation in the operation by adding an internal code. Shokrollahi (2006) use Raptor codes to get the internal coding range using a fixed distribution function. The Yin-Yang codes strategy (Ping et al., 2022) catches two sub-packets at a time, which corresponds to a constant degree of 2, where LT codes do not guarantee that all sub-packets of the original data participate in the coding. Online codes, Raptor codes, and Yin-Yang codes can guarantee that all sub-packets participate in encoding, but there is no guarantee that the redundant chain can cover all sub-packets, considering logical redundancy. Additionally, Yin-Yang code only encodes each sub-packet once, requiring 100% redundancy to cover all sub-packets if we add logical redundancy. LT codes still have a problem similar to YYC due to their small degree value. However, the RC has a larger degree, which means each chain contains more sub-packet data, making it possible to guarantee the addition of a small amount of logical redundancy to complete the decoding of information. To determine the logical redundancy that can cover all sub-packets, we use Gaussian XOR elimination to obtain the generated matrix. It is essential to note that this operation is performed only on the random matrix, and it is unnecessary to add droplets. That is, A ⊕ x = b, where only Gaussian XOR is performed on A. The generated matrix is screened to ensure the maximum decoding success rate and reduce the coding complexity.

As the algorithmic complexity of XOR or Gaussian elimination is K^3, we have used plasmids as DNA storage media in this experiment. Plasmids can accommodate longer DNA strands and provide more storage space, thereby effectively reducing the value of K for the same amount of data. This allows the encoder to run with lower algorithmic complexity.

The large degree average of RC provides an advantage in ensuring that the minimum redundant solution space covers all sub-packets after performing Gaussian precomputation, resulting in a maximum information decoding rate. This resolves the issue of insufficient sub-packet selection and low decoding rates commonly seen in fountain codes.

### 4.3 Screening for biological constraints

When the DNA sequence generated by the encoder fails to meet biochemical constraints, LT codes and Yin-Yang codes discard it and generate a new sequence. However, this approach leads to inefficiency as the vast majority of generated DNA sequences are discarded, reducing storage density. By using random equilibrium, we can proactively improve the DNA sequence’s biological constraints, thereby reducing the need for sequence discarding and increasing storage efficiency.

In the Fountain code system, some chains are assigned higher importance than others. For example, if a particular sub-packet is encoded into only one chain, then that chain is considered more important than others that have redundant information. Similarly, during the decoding process, a specific chain may be required to trigger the decoding, making it more important than other chains.

However, biological constraints can limit the efficiency of decoding, as important chains may fail to pass the biological constraints screening. This can result in overall low decoding efficiency. To overcome this issue, random equalization can be used, which assigns equal importance to all chains. This approach helps to ensure that important chains are not lost due to biological constraints, leading to better decoding efficiency.

Adding random equalization bits to the Fountain code system results in a decrease in overall information density, but it has the advantage of reducing the encoded address space. Previous studies have compensated for discarded chains that did not pass biological constraints by expanding the encoded address space to generate more droplet data. For instance, the Erlich team (Erlich and Zielinski, 2017) used 16 nt (4 bytes) of coding space to store seed information. However, as a vast majority of the generated droplet data was discarded, a total of 72,000 oligonucleotide sequences were generated, which utilized less than 0.00168% of the seed space.

In contrast, in this study, 25 chains were encoded with a Times space of 6 nt, resulting in an information space utilization of 6.1%, which will increase as the chain length increases. Therefore, it can be observed that adding random equalization significantly helps to save address space, which is one of the ways to improve information storage density.

Biological constraints have led previous scholars to attempt to regulate the encoding of binary data using code tables. These code tables are designed to transcode binary data into base data using specific rules that can help balance factors such as GC content and homopolymers (Ping et al., 2022)^.^ (Cao et al., 2022)^.^ (Zhang et al., 2020). However, the use of these code tables can lead to a reduction in information storage space and the creation of additional constraints during decoding due to their specialized design.

Random equalization has the advantage of preserving the optimal solution obtained by Gaussian XOR elimination, even in the presence of unbalanced homopolymer or GC content. Compared to previously reported methods such as fountain codes and yin-yang codes which discard the solution when they fail, this scheme improves the stability and decoding success rate of DNA strands even further. In the future, it is inevitable that long DNA double strands will be used for biological storage, maximizing the material advantage of DNA and fully utilizing its super high information storage density. However, as chain length increases, biological constraints become more difficult to satisfy. The use of stochastic equalization makes it possible to meet biological constraints for DNA strands of any information condition and length. Some research has been done on information equalization and encryption of long DNA chains.

## Data Availability

The datasets presented in this study can be found in online repositories. The names of the repository/repositories and accession number(s) can be found in the article/Supplementary Material.
