# Supplementary material for: Towards long double-stranded chains and robust DNA-based data storage using the random code system
Source: Front Genet. 2023 Jun 13;14:1179867. doi: 10.3389/fgene.2023.1179867 (PMC10294226; doi:10.3389/fgene.2023.1179867)
Supplement: Supplementary file 3 [file DataSheet1.docx]

Supplementary Material

Towards long double- stranded chains and robust DNA-based data storage using the random code system

Xu Yang^1†^, Xiaolong Shi^1^*, Langwen lai^1†^, Congzhou Chen^2^,huaisheng Xu^1^, Ming Deng^1^

*** Correspondence:**Xiaolong Shi
[xlshi@gzhu.edu.cn](mailto:xlshi@gzhu.edu.cn)

# RC Encoder

1 ) Determine the coding space, the coding space is mainly based on the synthesis technology of the DNA synthesizer as well as the budget, the total length of the DNA strand synthesized in this paper is 700nt, where the base allocation of the coding part is: a=20nt, t=6nt; p=639nt; Xe=10nt; Xee=2nt; XC=3nt;

2) Data partitioning, divide file into n chunks (n=S//P+1=14695//639+1=23), each of the chunks’ length is p nt (639nt);

3) Generate random vectors, using the random function in Python, to generate the range of (0, 2^n^ ) integer, while recording the number of times the random generator generates, the generated integer is transformed into an n-bit binary data vector, whose elements from the low (0) to the high (n-1) are 1 or not, indicating whether the chunks of L1, L2 ......Ln of the original file L partitioned in step 2) are involved in the encoding;

4) Use Gaussian XOR elimination algorithm to get the generated matrix, which has a higher decoding rate; Assume that random matrix are G and the storage redundancy is m. To ensure the robustness of the final storage content, firstly, random selected (n+m) strands, and then randomly selected n strands for 1000 times , and Gaussian elimination method is used to solve the matrix, we need to ensure that the elements on the main diagonal of the generated matrix obtained at least 550 times are all 1.

5)According the generated matrix, with the position of element to obtain the corresponding droplet;

6)The number of times the pseudo-random number generator is recorded and stored in the Times location, the Droplet obtained by step 5) is stored in the Data payload location.

7) The obtained packs are balanced, firstly, the conditions are screened, requiring homopolymer (no more than 3 consecutive bases), GC content (45%-55%), whether the primers are duplicated in the information coding space, if the DNA strand passes the screening, the XE position is marked as 0, and this packs is used as usable strand for storage; if the packs do not pass the screening, the packs are equalized and the Adapter sequence is used as the seed of the random generator to generate k times random bases of length (t+p)nt, and this random base is hetero- oriented with the bases of the packs that do not pass the screening to obtain a new base sequence, and the equalization is repeated continuously until the base sequence passes the screening or the XE equalization bit storage space is exhausted. For the strand that passes the screening after k equalization, store k in XE bit and record the base sequence of packs that pass the equalization at the same time.

8) Re-equilibration, due to the addition of the 10nt Xe equilibration bit, the Xe bit itself will also cause problems such as too high or too low homopolymer and GC content, we introduced a 2-nt re-equilibration bit, by re-equilibrating the XE bit, the equilibration method is the same as the equilibration method for Packs in 7), after re-equilibration, the equilibration times are stored in the XEE bit, Packs and XE bits are stored as the equilibrated The strands that do not pass the rebalancing are discarded directly.

9)Do the xor operation in xc bits for each strand, the result stored in XC;

10) Synthesis the obtained strands and final DNA storage.

# Decoder Decoding

1) The DNA strand obtained by sequencing is extracted according to the forward and reverse Adapter, and the proposed DNA strand length is judged and analyzed, if the length is lower or higher than the target length, a deletion/insertion error has occurred and this strand is discarded.

(2) If the sequencing strands’ length is correct, Do the xor operation in xc bits for each strand, if the result not consistent with the data stored in XC, it means that a substitution error occurred, discard this chain.

(3) If the number of chains completed by sequencing K> =n, the formal decoding process can be started, firstly, the information recovery, the primer information, as the seed of the random generator, is injected into the pseudo-random generator, and the information recovery is performed on the XEE, and then the information of the pseudo-random generator generation times of the XEE obtained after the recovery is used to recover the Times and data payload bits for data recovery to get the original data information.

4) Generate the corresponding 0-2^n^ random integers according to the generation times of the pseudo-random number generator corresponding to Times, and transform them into n-bit binary vectors to form a K*n-dimensional binary distribution sequence.

5) The data in the Data payload, converted from bases to decimal data, isotropic distribution sequence matrix constitutes the augmentation matrix, and the matrix is solved using the heterogeneous or Gaussian elimination method. (The solution rules are as follows: firstly, the matrix of order K, D, is combined with the Data matrix of K rows and 1 column to construct the augmented matrix, next, a judgment is made along the diagonal of the matrix (i from 0-k), if D[i][i]=1, all sequences under it are judged along the column, and if D[j][i]=1, all data in row i are dissimilar or with all data in row j. If D[i][i]=1, all data in row i are dissimilar or with all data in row j. If D[i][i]=0, then search down the column, find D[j][i]=1, swap two rows, then search down again, if there is still D[j][i]=1, then use the i row with the j row for the ensure the construction of an upper triangular matrix, the region below the diagonal of the matrix is all 0. Then, in accordance with the previous step, reverse the operation, the diagonal above the 1 all eliminated to 0, get the unique S1......Sk, and Data1......Datak, and convert this part of the solution into binary sequence, and then into base sequence, to get ChunksL1 and L2......Ln.

6) Complete the decoding of stored file data.
